# Stakeholder perceptions of the WHO country offices in Africa: implications for organisational reforms

**DOI:** 10.3389/fpubh.2025.1542835

**Published:** 2025-09-08

**Authors:** Olushayo Oluseun Olu, Abdulmumini Usman, Ndoungou Salla Ba, Patrick Kabore, Bei Achu, Mohammed Kakay, Hyelni Kulausa, Egide Rwamatwara, Joseph Okeibunor, Emmanuel Chanda, Alex Gasasira, Joseph Cabore

**Affiliations:** ^1^World Health Organisation Regional Office for Africa, Brazzaville, Republic of Congo; ^2^World Health Organisation Country Office, Kabul, Afghanistan

**Keywords:** stakeholders’ analysis, stakeholders’ perception, organisational effectiveness, organisational reforms, regional governance, WHO country office, health system accountability, World Health Organisation African Region

## Abstract

**Introduction:**

Evidence from internal audits and other evaluation reports shows that the World Health Organisation Regional Office for Africa and its country offices have had varied relationships with their stakeholders, including governments, donors, non-governmental organsations, and the United Nations. As part of a wider organisational reform, a stakeholder perception study was conducted to understand the insights of the organisation’s stakeholders on the performance of its country offices.

**Methods:**

We assessed stakeholders’ perceptions, expectations, and recommendations regarding the World Health Organisation African Region country offices using a self-administered questionnaire, conducted over multiple intervals from 2017 to 2020. Forty out of the forty-seven countries of the region were selected and included in the study. Respondents were purposively selected from the organisation’s key stakeholders in each country.

**Results:**

Responses were received from 865 respondents from 40 countries, representing a 100% overall country response rate. Governmental institutions, UN agencies, NGOs/civil society, donors, and others constituted 35% (303), 25% (216), 22% (190), 11% (95), and 6% (52) of the respondents, respectively. Twenty-six percent (225) of the stakeholders considered the ability of the World Health Organisation African Region country offices to manage threats as fair or poor. They were unaware of the organisation’s core functions, particularly the function of shaping the research agenda and articulating evidence-based policy options. Regarding the accessibility/technology and timeliness of how the organisation communicates public health information, 38% (329) and 34% (294) of stakeholders, respectively, rated the organisation fairly and poorly. The majority of partners identified health system strengthening, communicable and non-communicable diseases prevention, emergency preparedness and response, immunisation, and polio eradication as the top five areas for the organisation to focus on at the country level. In general, many of the respondents would like to see improvements in the quality of the organisation’s technical assistance, better integration into the wider United Nations system, and better recognition of and support to civil societies. The donors (25%) were the most critical of the organisation.

**Conclusion:**

We recommend a comprehensive organisational reform programme to address the negative perceptions identified in this study and reinforce the positive findings.

## Introduction

Historically, some stakeholders have perceived the World Health Organisation (WHO) as having become increasingly inefficient, bureaucratic, and unaccountable as it has grown (1–3). The six regional offices of the organisation have been viewed as stumbling blocks rather than championing the global health agenda in their regions (4). Gostin et al. observed that “excessive regionalization” of the WHO and the autonomy of the WHO regions have rendered the organisation unable to speak with one voice and to collectively implement global health policies (5). Other schools of thought believe that the WHO Regions are more interested in regional politics than advancing the global public health agenda, which tends to undermine the organisation’s headquarters in Geneva (6). The WHO African Region (WHO/AFR) has always been at the receiving end of these criticisms of underperformance, with its leadership heavily criticised for poor performance in 2013 (4). These criticisms grew in intensity following the West Africa Ebola virus disease outbreak (7–9). The organisation was widely blamed for the late and weak response to the outbreak, culminating in the long duration and high morbidity and mortality associated with it (10).

Unpublished internal audits and evaluation reports indicate that the WHO Regional Office for Africa (WHO/AFRO) and its Country Offices (COs) have had mixed relationships with their stakeholders. Whilst some stakeholders, particularly member states (MSs), generally perceived the organisation as adequate, others have raised concerns about its inability to provide comprehensive and timely technical assistance and public health leadership to its stakeholders. Funders have raised issues of inefficient use of donor funds, late reporting, and poor quality of donor reports as challenges that the organisation should address. Hence, there have been several calls for reforms of the WHO, particularly the WHO/AFRO and its COs (11, 12). WHO has undergone multiple reform initiatives over the past decades, although these efforts have largely been concentrated at the global level (13). Between 1989 and 1998, several attempts were made to address persistent challenges, including suboptimal performance at the country level, a gradual drift from the organisation’s core normative functions, and systemic management deficiencies. However, these efforts yielded only limited success (13). Subsequent reforms implemented between 1998 and 2003 aimed at internal restructuring and enhancing coherence between the six regional offices and the WHO headquarters, similarly achieved modest outcomes (13, 14). From 2010 onwards, the organisation embarked on additional reform agendas, focusing on improving governance, enhancing transparency, and increasing organisational effectiveness (13, 15). Nonetheless, these initiatives did not explicitly address the unique contextual and operational challenges of WHO/AFR, which remains one of the least targeted in prior reform efforts.

Thus, in 2015, the new WHO/AFR leadership established a major reform programme after identifying five key priorities, amongst which was transforming the organisation into a more effective, accountable, and results-driven agency. In consultations with its stakeholders, the new leadership outlined the modalities for implementing this priority in a document titled *“The Transformation Agenda of the World Health Organisation Secretariat in the African Region. 2015–2020*” (16). The Transformation Agenda (TA) had four focus areas, namely: (1) pro-results values, (2) smart technical focus, (3) responsive strategic operations, and (4) effective communications and partnerships. To ensure the effective implementation of the TA initiative, it was essential to capture the perceptions and expectations of WHO/AFR’s key stakeholders. The primary rationale was to generate insight into stakeholder views regarding the performance of the COs, which would, in turn, inform their functional review. This need became particularly pressing in light of the growing criticisms directed at the organisation in recent years (1–12, 17) and the existence of theories which highlight the importance of stakeholder engagement in organisational management (18). Freeman et al. described stakeholder engagement as an important tool for conceptualising and understanding organisations in the fields of strategy and management (19). Franklin et al. identified elements such as representativeness, transparency, accessibility, responsiveness, accountability, and sustainability as values that can be produced from stakeholder engagement (20).

Whilst several United Nations (UN) agencies have assessed their stakeholders’ perceptions as part of wider organisational evaluation programmes (21–23), to the best of our knowledge, no prior study has systematically and specifically examined the stakeholder perceptions of WHO/AFR and its COs. This article, therefore, presents empirical evidence from a stakeholder perception study conducted in the WHO/AFR between 2017 and 2020. The findings discussed herein are derived from a quantitative and, to a certain extent, qualitative analysis of stakeholders’ views on the performance, expectations, and areas for improvement of COs in the WHO/AFR. The survey is underpinned by the conceptual framework of how stakeholder perceptions and engagement can catalyse or inform public health organisational reforms. The framework drew from the foregoing discourse, particularly the perceived mixed relationship between WHO and its stakeholders, health systems thinking, change management theories, and participatory governance models (18–20, 24, 25). This framework comprises five critical steps namely: (1) stakeholder mapping based on analysis of their perceived opinions; (2) stakeholder engagement through policy dialogue, perception surveys, and consultations; (3) integration of stakeholders’ feedback into organisational reform planning processes through policy briefs and other documents; (4) implementation of organisational reforms; and (5) monitoring, evaluation and feedback of the lessons learned into the organisational reform processes.

## Materials and methods

### Study design

We conducted a cross-sectional study to assess the stakeholders’ satisfaction with the WHO/AFR COs’ performance of the organisation’s core functions in the Region. Data collection was conducted over multiple intervals from 2017 to 2020.

### Study setting and participants

The WHO/AFR, comprising 47 MSs^1^ predominantly in sub-Saharan Africa, continues to face major public health challenges despite progress in immunisation and disease control. In 2021, its Universal Health Coverage (UHC) index was 44/100, significantly below the global average. The Region also reports high maternal and child mortality rates and a substantial burden of infectious and non-communicable diseases. Health system capacity remains weak due to poor governance, underfunding, and conflict, with government health expenditure at 9.8% in 2018, significantly below the Abuja target of 15%. The low health workforce density further constrains service delivery. The WHO/AFR, through its 47 COs, supports its MSs to address these challenges through the implementation of its core functions such as providing technical assistance, adapting health standards, and coordinating health sector partners (26). These are conducted in collaboration with a range of bilateral and multilateral health development and humanitarian stakeholders, including national ministries of health, UN agencies, international and national non-governmental organisations, civil society actors, and donor agencies. To effectively do these, the COs are organised into programmatic areas such as health system strengthening (HSS), communicable diseases control (CD), non-communicable diseases control (NCD), emergency preparedness and response (EPR), maternal and child health (MCH), immunisation, polio eradication, amongst others. The study participants were drawn from this broad spectrum of health development stakeholders, including Ministers of Health or their designees, heads of health development cooperation within donor agencies, country directors of international and national non-governmental organisations (NGOs), and heads of relevant UN agencies.

### Questionnaire development and validation

#### Theoretical framework

The theoretical basis for the development of the questionnaire was the objectives and operational guidelines of the TA and the conceptual framework that underpinned this study. To ensure conceptual consistency, the questions were designed to reflect and test the stakeholders’ perception of the organisation’s core functions and performance. This theoretical framework facilitated the systematic design of the questionnaire, enabling it to assess the perceived contributions and operational effectiveness of the COs.

#### Questionnaire design

The questionnaire comprised 15 questions, 12 closed-ended and 3 open-ended. The closed-ended questions used 3–4-point Likert scales to gauge levels of agreement or satisfaction across the key thematic areas. The open-ended questions were designed to capture qualitative perceptions, offering respondents an opportunity to elaborate on their experiences and provide context-specific recommendations. The questionnaire covered thematic areas such as the stakeholder awareness of WHO’s core functions; satisfaction with the technical assistance provided by the COs across these functions; perceptions of the COs’ capacity to address public health threats; and evaluation of communication methods and engagement strategies employed by the COs, amongst others (Supplementary File 1).

#### Validation process

The draft questionnaire was reviewed by members of the TA implementation team and other WHO/AFRO technical officers with extensive experience in regional and country-level operations. The reviewers evaluated the questions for clarity, relevance, and alignment with the TA and organisation’s core functions. Subsequently, the questionnaire underwent pilot testing in four countries, namely Togo, Senegal, South Africa, and South Sudan. Feedback from the review and pilot was used to refine and finalise the questionnaire. The final version of the questionnaire was translated into French and Portuguese to accommodate respondents’ preferences.

### Sampling and data collection

**Figure 1:**
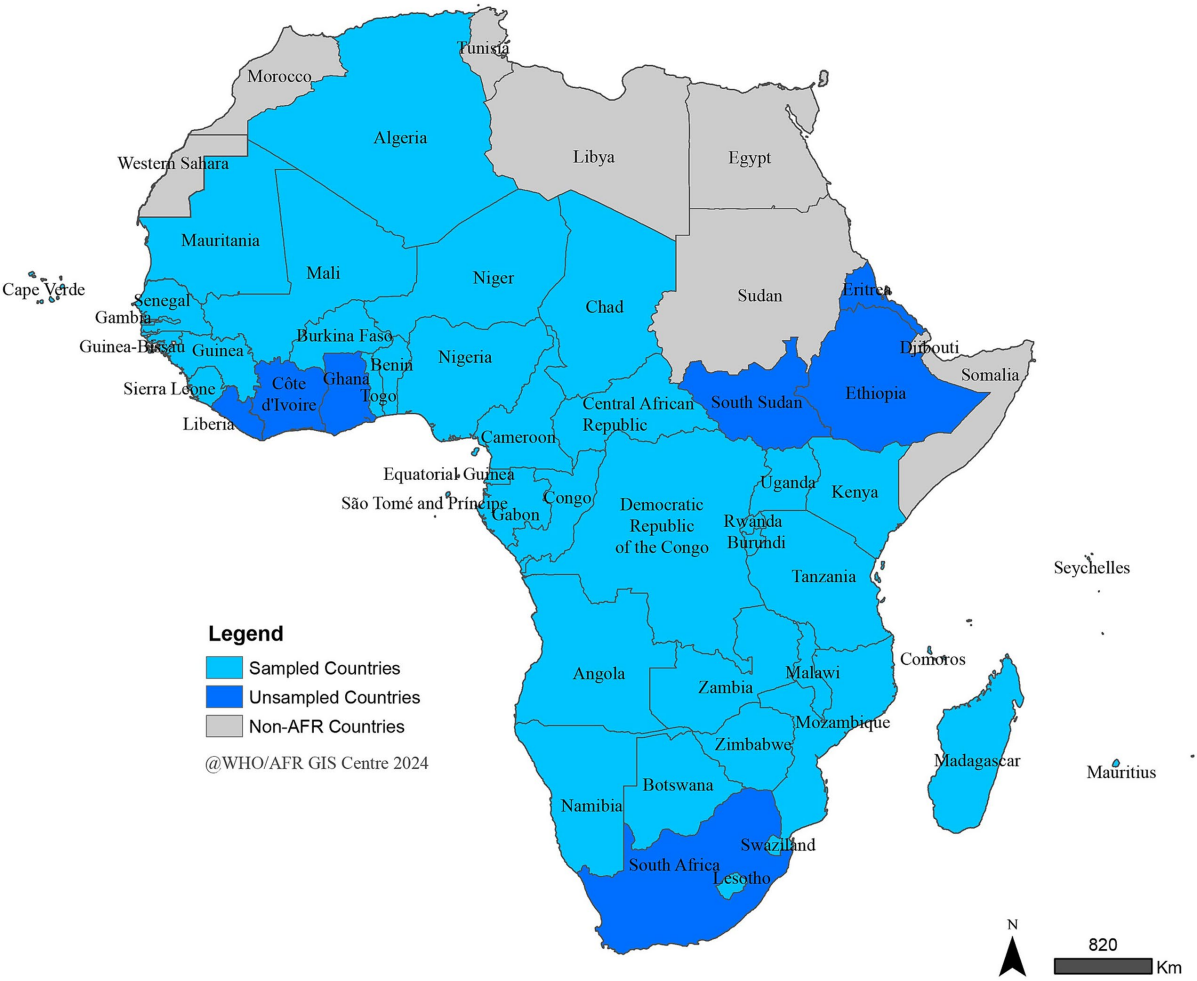
WHO/AFR member states sampled for the stakeholders’ survey.

A total of 40 out of the 47 MSs in WHO/AFR, representing the Region’s three official languages (English, French, and Portuguese) and its four major geopolitical sub-regions: Western, Central, Eastern, and Southern Africa (Figure 1), were included in the study. Seven COs were excluded from the study because the study started after the completion of the review of those countries. Within each country, a purposive sampling approach was employed to identify and recruit key stakeholders and institutional partners of the COs. Data were collected via a single-use web link to a SurveyMonkey database. The link was electronically shared with all identified respondents, who were invited to self-complete the survey.

### Data analysis

A member of the research team regularly monitored the database to ensure data completeness and quality. The data were subsequently cleaned and exported into Microsoft Excel for analysis. Descriptive statistics were performed on the closed-ended questions, with frequency distributions calculated and presented in graphical form. For the open-ended questions, a narrative analysis approach was employed. The responses were transcribed and independently reviewed multiple times by two members of the research team to identify the salient ideas, particularly those aligning with or expanding upon the findings from the quantitative data. Key messages and thematic insights were then extracted, taking into consideration the context in which the responses were provided. These ideas were organised into thematic categories and synthesised into narrative summaries, which were presented as illustrative quotes.

### Ethical consideration

Ethical approval for the study was sought from the WHO/AFRO Ethics Review Committee. The Committee granted exemption from formal ethical clearance, as the study was conducted within the context of an internal organisational reform initiative and posed no foreseeable risk to the human participants. To safeguard confidentiality and privacy, no personal identifiers and information were collected, thereby ensuring the anonymity of respondents.

## Results

Responses were received from 865 respondents from 40 countries, representing a 100% overall country response rate. Governmental institutions, UN agencies, NGOs/civil society, donors, and others, respectively, constituted 35% (303), 25% (216), 22% (190), 11% (95), and 6% (52) of the respondents. Cameroon had the highest response rate of 92 (11%) responses, followed by Nigeria (74, 9%), Tanzania (66, 8%), Malawi (58, 7%), and Niger (50, 6%). Sierra Leone had a 42 (5%) response rate, whilst Mauritius, Mali, and the Central African Republic each had a 4% response rate. The lowest response rate of 2% was received from Benin, Lesotho, and Mauritania.

### Awareness of the core functions of the WHO COs

**Figure 2:**
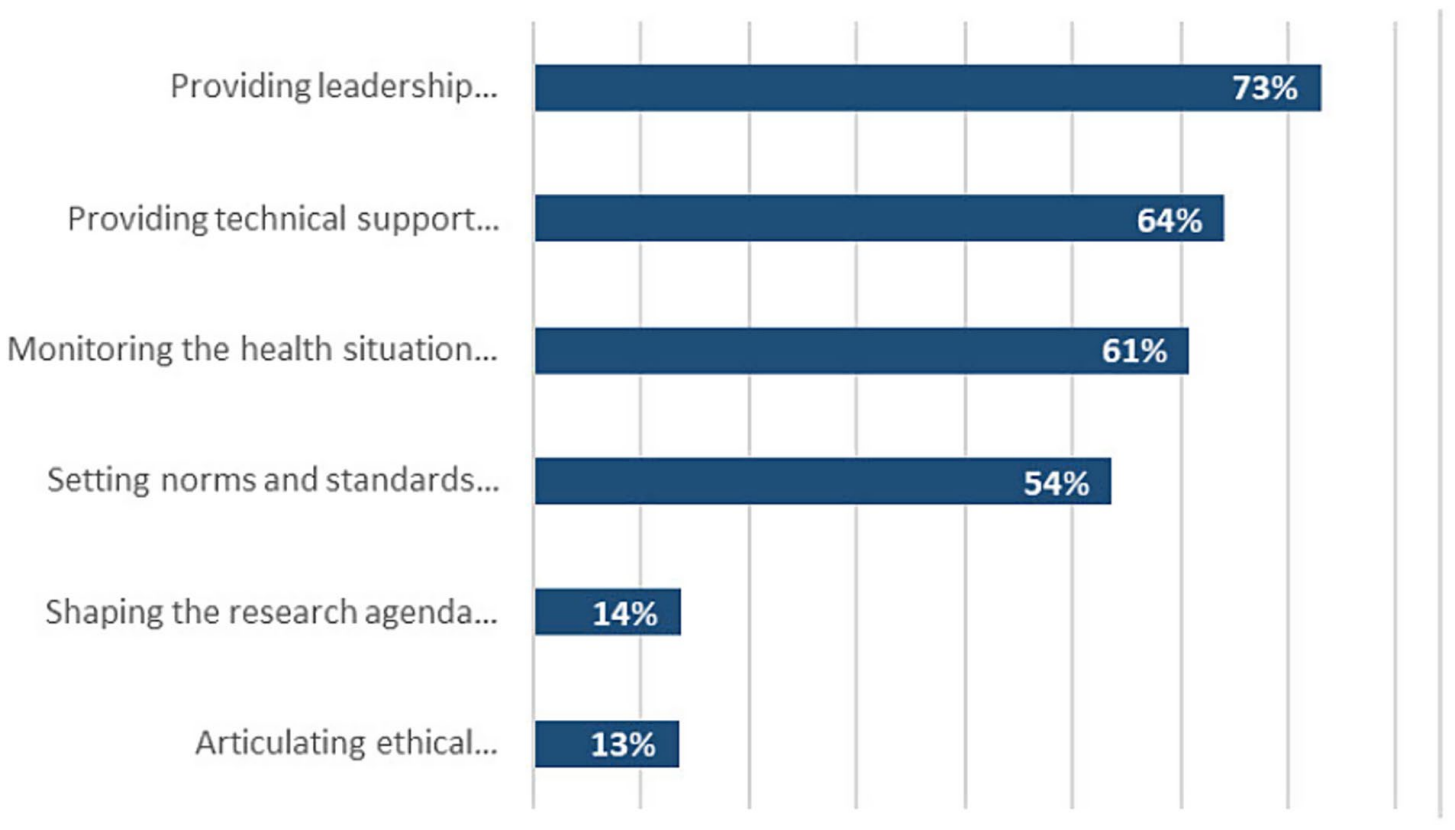
WHO/AFR stakeholders’ awareness of the WHO core functions.

Stakeholders were aware of 4 out of the 6 core functions of the WHO, with most of them unaware of the role of the WHO in setting the public health research agenda and articulating evidence-based policy options (Figure 2). The majority of stakeholders believed that the WHO/AFR COs were indispensable (35%) or important (53%) for the functioning of their organisation.

### Ability of the WHO COs to manage health threats

One-quarter (26%) of partners see the WHO/AFR COs’ ability to manage threats as fair or poor, especially the donors (43%). A donor stated “*I have seen WHO Geneva or AFRO support in countries during emergencies and I have confidence in them, I lack confidence in the country office’s ability and that may be because they just do not communicate well on what they are doing.”* Whilst a civil society stakeholder reported that *“their resource, including number of staff is limited effectively manage health threats in the country”* (sic).

### Confidence in the WHO/AFR COs

**Figure 3:**
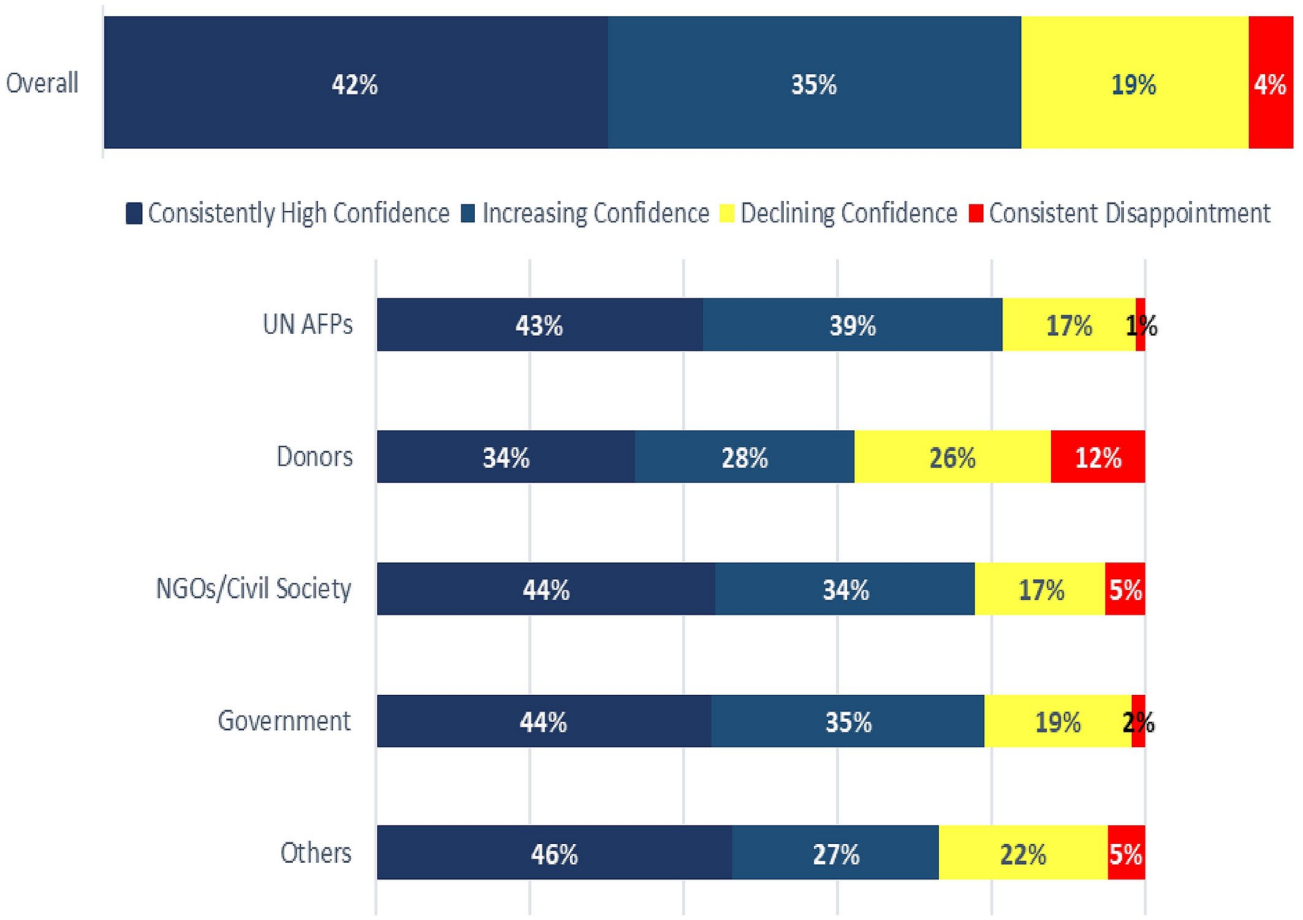
WHO/AFR stakeholders’ view/perception about the WHO country offices.

Approximately 19% of the respondents reported declining confidence in the COs over the last 2 to 3 years. Among donors, 26% had declining confidence, and 12% were consistently disappointed in the COs during the 2 years preceding the study (Figure 3). A donor said *“We do not see their presence at the MOH TWG, strategic and policy table”* whilst another commented that *“actions of the WCO are only marginally linked to country priorities; no capacity for a much-needed change management; many doubts on professional capacities of many of its staff.”* A civil society stakeholder commented that “*Challenging to perceive (WHO’s) role as supportive to Govt., some staff adopt a negative/ constant blame-game attitude with government.”* Twenty-five percent (25%) of donors said they would be critical of the WHO/AFR COs. A donor said “*WHO is not visible at the table.”*

### Satisfaction with the WHO COs’ technical assistance

**Figure 4:**
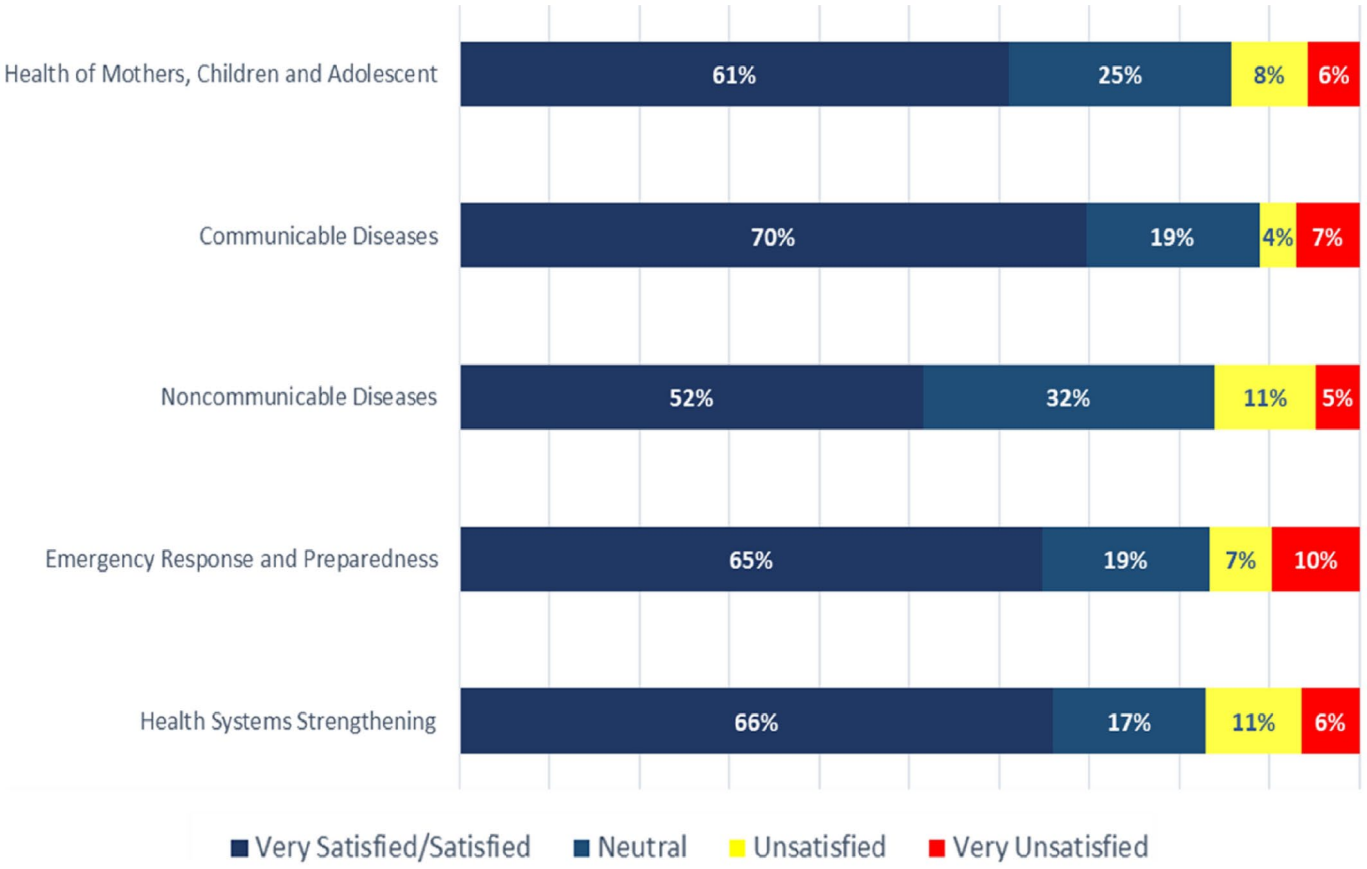
Stakeholders’ satisfaction with WCO’s technical assistance to programmes.

Satisfaction with the COs’ technical support ranged from 52% for NCDs to 70% for communicable diseases (CDs) (Figure 4). Donors (over 20%) were the least satisfied across all levels of the WHO’s technical support, especially for HSS, NCDs, and MCH. A UN stakeholder said, *“There is no tangible support from WCO to MOH in non-communicable disease. Even some WCO staff do not know who is WCO focal point for NCD.”* Whilst a donor said, *“They are very good at communicable diseases, providing both technical support and leadership. They can do a lot more in other areas.”*

The majority of partners identified HSS, CD, EPR, NCDs, and immunisation and polio eradication as the top five areas for the WHO to focus on at the country level. More than half of the donors also mentioned health information management. A government stakeholder said, “*Non-communicable diseases are claiming more and more lives in the world…”* whilst another said, *“It’s important for WHO to continue efforts to support countries build systems, including coordination for effective and efficient service delivery.”*

### Satisfaction with the WHO COs’ method of communication

Over 60% of respondents considered WHO/AFR COs to be effective at influencing policies to improve people’s health and well-being. Regarding the accessibility, technology, and timeliness of how the WHO/AFR COs communicate public health information, 38% and 34% of stakeholders, respectively, rated the organisation fair and poor. A government stakeholder said that “*Much of public health information from WHO is on the internet; the problem for my country is that internet connectivity and use are low,”* whilst a civil society stakeholder said, *“Mostly WHO uses websites. Use of local media, such as radio, would help reach people in rural areas as well those who are in majority.”* The respondents would like WHO/AFR COs to continue their roles in advocacy, technical assistance to governments, leadership, health coordination, and public health communication, whilst asking the COs to stop duplicating the efforts of other partners, direct implementation of projects, yielding to government pressure, delayed reporting, and non-collaborative decision-making.

Donors were the most critical of the WHO, with 25% expressing their dissatisfaction with the organisation. Approximately 17% of other partners were also critical of the organisation when asked. Donors would like the organisation to improve its capacity to coordinate and provide health information and better donor relations. In general, government stakeholders mostly appreciated the COs’ work but would like to see improvements in the quality of the organisation’s technical assistance. UN agencies also appreciated the work of the COs but requested better integration into the wider UN system. Civil societies and NGOs felt neglected by the COs and sought better guidance and recognition from the organisation.

## Discussion

Stakeholder engagement is a critical determinant of organisational effectiveness, sustainability, and success. It facilitates trust building, enhances institutional reputation, improves service delivery, aligns organisational actions with the expectations of those affected by or influencing the organisation, and contributes to overall performance optimisation. Furthermore, understanding stakeholders’ perceptions is essential for identifying their needs, fostering commitment and ownership, and generating evidence to inform strategic decision-making and reform processes (27). Against this backdrop, this study sought to assess stakeholder satisfaction with the performance of the WHO/AFR COs. The findings indicate that whilst stakeholders generally held favourable views regarding the role and contributions of the WHO/AFR COs, there was limited awareness of some of their core functions, particularly in relation to research agenda-setting and their facilitative role in policy dialogue.

The limited awareness of the organisation’s core functions and its public health information products is not unexpected. The primary channel for disseminating such information, its official website, seems to be suboptimal, particularly given that some stakeholders report limited or no engagement with the organisation’s online platforms. This highlights the need for more innovative and context-appropriate communication strategies to enhance the visibility and accessibility of the organisation’s mandate and public health outputs (28). Closely linked to this issue is a reported decline in stakeholder confidence, which may be attributed to several factors. First, the perceived absence and limited visibility of the organisation and its personnel at key national and regional forums, as noted by several partners, may have been interpreted as a lack of engagement or seriousness. Second, the conduct and interpersonal approach of certain staff members may have inadvertently conveyed unfavourable impressions to stakeholders, further undermining trust in the organisation (29). For instance, a civil society stakeholder said, “*Depending on the area of discussion, WHO country office does excellent work, while in some area where you expect them to be present, you do not have them visible.”* Whilst another said, “*the office (WHO) needs to come closer to National NGOs.”* Third, inadequate engagement and inclusion of its stakeholders in planning its health programmes may be another reason (27, 30).

The study showed that the WHO/AFR COs’ stakeholders are placing increasing demands on the organisation despite acknowledging its limited financial resources and inadequate staffing levels. A government stakeholder said, “*The WHO country office works hard. I think it tries its best within a very challenging environment. They just need to adapt quickly and mobilise more funds to enhance more health-critical domains.”* Whilst a UN partner highlighted that “*I would say that they are not always very helpful, maybe due to the limited number of staff, who are often deployed. Yet, I am sometimes satisfied.”* This finding is consistent with previous literature, which has attributed the organisation’s underperformance to insufficient funding, inadequate staffing, and a lack of clear prioritisation of its programmes (17).

The belief of many stakeholders that the organisation has focused more on CDs at the expense of NCDs is not surprising. Stuckler et al. documented that 87% of the WHO’s (including WHO/AFR’s) financial resources were allocated to CDs, with only 12% to NCDs. They also observed a misalignment in the WHO resource allocation and the health needs of its beneficiaries (31). This underscores the need for the organisation to reprioritise its work based on the most critical health challenges and the needs of its MSs vis-à-vis its financial and human resource capacity. Furthermore, the strategic recruitment of versatile and multi-skilled staff would enhance the organisation’s ability to effectively fulfil its obligations to stakeholders.

The finding that donors appear to be the most critical amongst the stakeholder groups may partly explain the persistent funding constraints currently facing the organisation (32, 33). This is a matter of significant concern that requires urgent and comprehensive attention to restore donor confidence and improve the resourcing of the WHO/AFR COs. The declining stakeholder confidence observed in this study may be attributed to several interrelated factors identified during the study, including insufficient visibility and communication of the organisation’s work, perceived deficiencies in staff engagement and responsiveness, and limited institutional capacity to effectively deliver on core mandates.

The preceding findings are comparable to those of similar studies of other UN offices. An evaluation of the United Nations Development Programme office in Nigeria and Uganda identified concerns about implementation delays and inefficiencies in its management of programmes and resource mobilisation (34, 35). Similar findings were also observed in the evaluation of the United Nations Children Fund in Sri Lanka (22) and in a health stakeholder analysis conducted in Kenya (29).

The study findings should be interpreted against the backdrop of some limitations. First, the use of a self-administered questionnaire in an uncontrolled setting may have introduced response bias, a known limitation of this data collection method. Second, the exclusion of seven countries could have resulted in selection bias, thereby limiting the generalisability of the findings to the entire African region. Third, the study and analysis were primarily descriptive and did not employ inferential statistical methods, which restricts the ability to draw definitive conclusions regarding associations or causal relationships between variables. Fourth, the use of purposive sampling for the selection of the respondents could have introduced some biases that a random sampling would have eliminated. Nonetheless, these limitations are partially mitigated by the study’s relatively large sample size and the inclusion of 85% of countries in the WHO African Region, which enhances the representativeness and robustness of the findings.

## Conclusion

Despite generally favourable stakeholder perceptions of the WHO/AFR COs, several negative views persist that may undermine the organisation’s credibility and effectiveness. These reveal major issues about how the organisation’s performance is viewed, with implications for the visibility, level of trust, and funding by its stakeholders, particularly the donors. Thus, urgent steps are required to reform the organisation by consolidating the positive findings and mitigating the negative perceptions from this study.

First, key organisational reform priorities should include enhancing the visibility and communication of the WHO/AFRO’s mandate and public health outputs to both stakeholders and the wider public. Such efforts should prioritise interventions, which would ensure more visibility in the national development aid and policy spaces and enhance stakeholder inclusion in the decision-making process of the organisation. Second, strengthening the organisation’s capacity to deliver high-quality, evidence-based technical assistance through an expansion of its technical expertise base and investing in staff development, motivation, and performance management would also be critical in addressing the negative views of the organisation. Third, the reform agenda should incorporate transparent, evidence-based mechanisms for setting strategic priorities to ensure alignment with the needs of its MSs and emerging public health challenges.

Fourth, of particular urgency is the need to establish clear frameworks for constructively engaging the organisation’s donors to address long-standing negative perceptions that may be impeding resource mobilisation. Open, sustained dialogue and responsiveness to donor feedback are essential to rebuilding trust and securing sustainable funding. Fifth, the establishment of key managerial and administrative performance indicators would also be required to regularly monitor the organisation’s performance in addition to periodic stakeholder perception surveys.

Finally, against the backdrop of the stakeholders’ expectations, structural reforms of the organisation’s staffing model are required to ensure that the right quality and quantity of staff are recruited and strategically placed in all COs. Collectively, these measures would strengthen the organisation’s operational effectiveness and institutional credibility, thereby strategically enhancing the capacity to support MSs in achieving global and regional health, development, and humanitarian goals. However, common challenges to organisational reforms, such as inadequate planning, resistance from staff, and resource constraints, should be anticipated and proactively addressed to facilitate the successful implementation of reforms.

## Data Availability

The original contributions presented in the study are included in the article/Supplementary material, further inquiries can be directed to the corresponding author.

## Footnotes

^1^Algeria, Angola, Benin, Botswana, Burkina_Faso, Burundi, Cameroon, Cape_Verde, Central_African_Republic, Chad, Comoros, Ivory_Coast, Democratic_Republic_of_the_Congo, Equatorial_Guinea, Eritrea, Ethiopia, Gabon, The_Gambia, Ghana, Guinea, Guinea-Bissau, Kenya, Lesotho, Liberia, Madagascar, Malawi, Mali, Mauritania, Mauritius, Mozambique, Namibia, Niger, Nigeria, Republic_of_the_Congo, Rwanda, São_Tomé_and_Príncipe, Senegal, Seychelles, Sierra_Leone, South_Africa, South_Sudan, Eswatini, Togo, Uganda, Tanzania, Zambia, Zimbabwe.
