# Supplementary material for: Stakeholder perceptions of the WHO country offices in Africa: implications for organisational reforms
Source: Front Public Health. 2025 Sep 8;13:1542835. doi: 10.3389/fpubh.2025.1542835 (PMC12450994; doi:10.3389/fpubh.2025.1542835)
Supplement: Supplementary file 1 [file Data_Sheet_1.pdf]

Welcome to the Partner Perception Survey

Thank you for participating in our survey. Your feedback is important.

As part of the Transformation Agenda, approved at the 65th Session of the Regional Committee of WHO in the AFRO region, WHO Country Offices are undergoing functional reviews to ensure better alignment of the workforce and operations of WHO and host countries' health situation, needs, and priorities. The outcome of the implementation of the functional reviews is for the Country Office to deliver high-quality technical advice and operational support relevant to and appreciated by the host government and international and national partners.

The operational methodology of the Functional Review consists of establishing the structure of a country office as a result of a multi-level consultative process. Consultations are conducted with the Government, UN Agencies, NGOs, civil societies, bilateral and multilateral organizations. This is followed by an extensive workshop with the country office to enable them to align their operations and structure to the strategic priorities while identifying required functions and expertise.

Within this background, the Functional Review Team is scheduled to review the WHO Country Office in the coming weeks. In such anticipation, it is of the utmost importance to get your views, opinions about the WHO Country Office in an effort to continuously provide technical support to the host country.

Thank you very much for taking the time to take this survey. The survey should take you 5-7 minutes. Be assured that the survey is completely anonymous.

Sincerely,
AFRO Country Functional Review Team

WHO Transformation Agenda - Country Functional Reviews

Profile

* 1. Please select in which country you are working

* 2. Please describe your organization using one of the criteria below

- Government
- United Nations Agency, Funds or Programs
- Non Governmental Organization (NGOs)/Civil Society
- Donor
- Other (please specify)

* 3. Which areas of WHO's responsibilities (core functions) are you most familiar with? (Please select up to three (3) boxes)

- [ ] Providing leadership on matters critical to health and engaging in partnerships where joint action is needed
- [ ] Shaping the research agenda and stimulating the generation, translation and dissemination of valuable knowledge
- [ ] Setting norms and standards and promoting and monitoring their implementation
- [ ] Articulating ethical and evidence-based policy options;
- [ ] providing technical support, catalyzing change, and building sustainable institutional capacity
- [ ] Monitoring the health situation and assessing health trends.

* 4. How essential is the WHO Country Office for your organization?

- ○ Indispensable
- ○ Important
- ○ Limited Importance
- ○ Irrelevant

* 5. Which of the following best describes your view of WHO's Country Office over the past two to three years?

- ○ Increasing confidence
- ○ Consistently high confidence
- ○ Declining confidence
- ○ Consistent disappointment

Please Comment

* 6. How satisfied are you with WHO country office technical support in the following areas?

	Very Satisfied	Unsatisfied	Neutral	Satisfied	Very Unsatisfied	N/A
Health System Strengthening	○	○	○	○	○	○
Emergency Response and Preparedness	○	○	○	○	○	○
Noncommunicable Diseases	○	○	○	○	○	○
Communicable Diseases	○	○	○	○	○	○
Health of mothers, children and adolescent	○	○	○	○	○	○

Please Comment

* 7. How does your organization view the WHO Country Office ability to manage health threats?

- ◯ Excellent
- ◯ Good
- ◯ Fair
- ◯ Poor

Please Comment

* 8. Which of the following areas of work below do you perceive to be the most important for the WHO Country Office to support? Please select your top five (5) choices.

- ☐ Increased access to key interventions for people living with HIV and viral hepatitis
- ☐ Adaptation and implementation of the global strategy and targets for tuberculosis prevention, care and control
- ☐ Increased access of populations at risk to preventive interventions, diagnostic confirmation of malaria and first-line antimalarial treatment
- ☐ Neglected Tropical Diseases
- ☐ Support the country in developing and implementing national multi-year plans and annual implementation plans, including micro-planning for immunization, with a focus on under-vaccinated and unvaccinated populations
- ☐ Increased access to interventions to prevent and manage noncommunicable diseases and their risk factors
- ☐ Country' capacity strengthened to develop and implement national policies, plans and information systems in line with the comprehensive mental health action plan 2013–2020
- ☐ Violence and injuries
- ☐ Disabilities and rehabilitation
- ☐ Country enabled to develop and monitor implementation of action plans to tackle malnutrition in all its forms and achieve the global nutrition targets 2025 and the nutrition components of the Sustainable Development Goals
- ☐ Reproductive, maternal, newborn, child and adolescent health
- ☐ Ageing and Health
- ☐ Gender, equity and human rights mainstreaming
- ☐ Country enabled to assess health risks and develop and implement policies, strategies or regulations for the prevention, mitigation, and management of the health impacts of environmental and occupational risks
- ☐ National health policies, strategies and plans
- ☐ Integrated people-centred health services
- ☐ Access to medicines and health technologies and strengthening regulatory capacity
- ☐ Health systems, information and evidence
- ☐ Alert and response capacities
- ☐ Emergency risk and crisis management
- ☐ Outbreak and crisis response
- ☐ Food safety
- ☐ Polio eradication
- ☐ Health Coordination

Please Comment

* 9. Which groups do you consider the most effective at influencing policy for improving people's health in this particular country? Please select your top three (3) choices.

- ☐ National Goverments
- ☐ Media
- ☐ WHO
- ☐ World Bank
- ☐ Global Funds
- ☐ Private Philanthropy
- ☐ European Union insuirtions
- ☐ GAVI
- ☐ UNFPA
- ☐ IOM
- ☐ Celebrities
- ☐ UNICEF
- ☐ Civil Societies
- ☐ NGOs

* 10. How do you rate the way in which the WHO Country Office communicates its public health information?

	Excellent	Good	Fair	Poor
Reliability/Accuracy	○	○	○	○
Usefulness	○	○	○	○
Timeliness	○	○	○	○
Accessibility/Technology friendly	○	○	○	○
In a language you can use	○	○	○	○

Please Comment

* 11. Which of the below phrases best describes how you speak about the WHO Country Office?

- ○ Positively without being asked
- ○ Positively when asked
- ○ Neutral when asked
- ○ Critical when asked
- ○ Critical without being asked

Please Comment

* 12. Do you trust the WHO Country Office to take the necessary measures to ensure the independence of its public health expert advisers?

- ◯ Always
- ◯ Most of the time
- ◯ Sometimes
- ◯ Rarely
- ◯ Never

Please Comment

* 13. What would you want WHO Country Office to continue todo?

* 14. What would want WHO Country Office to do more of?

* 15. What would you want WHO Country Office to stop doing?
